# Current and Future Perspectives on Alginate Encapsulated Pancreatic Islet

**DOI:** 10.1002/sctm.16-0116

**Published:** 2017-02-02

**Authors:** Berit L. Strand, Abba E. Coron, Gudmund Skjak‐Braek

**Affiliations:** ^1^ NOBIPOL, Department of BiotechnologyNTNU Norwegian University of Science and TechnologyTrondheimNorway

**Keywords:** Diabetes, Capsules, Encapsulation, Islets, Stem Cells, Alginate

## Abstract

Transplantation of pancreatic islets in immune protective capsules holds the promise as a functional cure for type 1 diabetes, also about 40 years after the first proof of principal study. The concept is simple in using semipermeable capsules that allow the ingress of oxygen and nutrients, but limit the access of the immune system. Encapsulated human islets have been evaluated in four small clinical trials where the procedure has been evaluated as safe, but lacking long‐term efficacy. Host reactions toward the biomaterials used in the capsules may be one parameter limiting the long‐term function of the graft in humans. The present article briefly discusses important capsule properties such as stability, permeability and biocompatibility, as well as possible strategies to overcome current challenges. Also, recent progress in capsule development as well as the production of insulin‐producing cells from human stem cells that gives promising perspectives for the transplantation of encapsulated insulin‐producing tissue is briefly discussed. Stem Cells Translational Medicine *2017;6:1053–1058*

**Significance Statement**

Transplantation of pancreatic islets in immune protective capsules holds the promise as a functional cure for type 1 diabetes. The present article discusses briefly important capsules properties such as stability, permeability, and biocompatibility, and possible strategies to overcome current challenges. Recent progress in capsule development as well as the production of insulin producing cells from human stem cells gives promising perspectives for the transplantation of encapsulated insulin producing tissue.

## Diabetes, Islet Transplantation, and the Principle of Encapsulation for Immune Protection

Diabetes is one of the leading causes of both morbidity and mortality worldwide and the number of diabetics are rapidly increasing 1. Type 1 diabetes, accounting for about 10% of the incidents of diabetes, is an autoimmune disease where the immune system attacks the body's own beta cells that produce the insulin needed to regulate the blood glucose. The disease is normally treated by daily insulin injections, however poor regulation of the blood glucose is associated with secondary complications such as cardiovascular disease, nephropathy, and neuropathy 1. Newer ways of distributing insulin exist, including infusions and injections; where continuous glucose monitoring allows tighter control over the blood glucose. However, an appealing alternative to controlling the blood glucose is via the transplantation of insulin‐producing cells. Pancreas transplantation from deceased donors is one alternative, however this requires major surgery followed by associated risks. Pancreatic islets, cell clusters containing the endocrine function of the pancreas, can be isolated from the pancreas through enzymatic digestion 2, 3. In the future, stem cells may offer an unlimited source of insulin‐producing cells. This is a field of rapid development, where exciting progress is made in the development of insulin‐producing cells both in vivo and in vitro 4, 5, 6.

**Figure 1:**
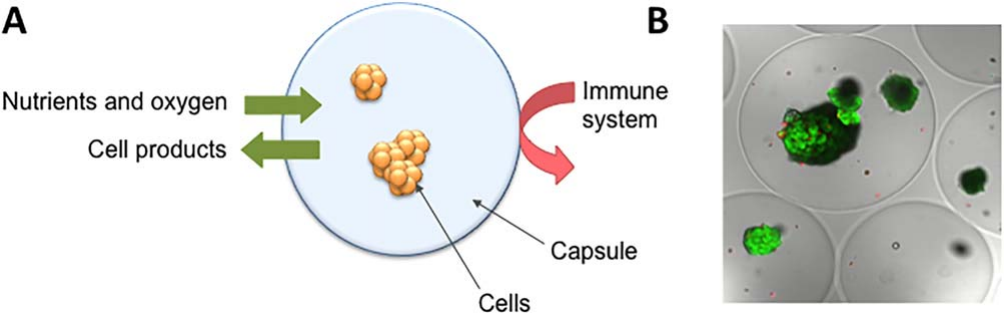
Immune isolation by encapsulation. Concept illustration of cells in capsule where nutrients and oxygen can diffuse into the capsule and cell products (e.g. insulin) can diffuse out, but effectors of the immune system is excluded **(A)** and picture of encapsulated human islets **(B)** stained by calcein and ethidium homodimer to visualize living and dead cells, respectively. The photomicrograph is a cross‐section obtained using confocal microscopy. Bead diameter is approximately 500 μm. Picture is printed from 8.

Today, pancreatic islets are transplanted with a limited surgical procedure via injection into the portal vein to the liver. Although being successful in reducing the need for external insulin and also in reducing the incidents of hyper‐ and hypo‐glycemic events, the transplantation of insulin‐producing cells requires the need for immune suppression to avoid graft rejection 7. The use of immune suppression is associated with increased risk of infections and cancer. However, islet transplantation is still considered a better alternative for patients with severely unstable blood glucose and in particular those previously transplanted with, for example, a kidney. Encapsulation of the pancreatic islets offers an alternative to immune suppression where the capsules act as semipermeable membranes that hinder the immune attack of the transplanted cells (Fig. 1). Importantly, the capsules must allow for the diffusion of oxygen and nutrients to the encapsulated cells and also permit the diffusion of insulin out to the surrounding environment.

The immune protection of pancreatic islets by using capsules was first demonstrated in rats by Lim and Sun 9. Capsules are advantageous over larger devices in that they provide rapid diffusion of nutrients as well as oxygen. In addition, the use of capsules reduces the risk of graft failure due to the distribution of cells in numerous devices, limiting the mechanical failure to only the affected device containing a limited portion of the transplanted graft. Also, the ease of transplantation by laparoscopy is attractive 10. The capsules are normally injected into the peritoneal cavity, where the encapsulated cells get the access to nutrients and oxygen from the surrounding fluids.

## Capsules for Immune Protection

Many different variations of capsules and capsule materials have been suggested for the immune isolation of pancreatic islets as reviewed in 11. Of polymers, agarose, chitosan, gelatin, polyethylene glycol, methacrylic acid, 2‐hydroxyethyl methacrylate have been used, however the vast majority is based on alginate 11. Alginate is a structural polysaccharide found in seaweed that is nontoxic, exhibiting a low immunogenic profile 12. It has the unique property of forming a gel at physiological conditions with divalent ions, such as calcium. The encapsulation is performed by dripping a mixture of alginate and islets into a solution of divalent ions, where the droplet is immediately fixed at the surface. The gelation proceeds throughout the droplet, resulting in a gel bead with entrapped islets that are kept viable and functional. In the original encapsulation procedure, the gel bead was further coated with a layer of polycation 9. Poly‐l‐lysine (PLL) 9, 13, poly‐l‐ornithine (PLO) 14 and poly‐methylene co‐guanidine 15 form stable complexes with alginate at the surface of the gel beads. The inner core of the gel beads can further be liquefied by using chelating compounds (e.g., citrate or EDTA), leaving the outer shell as the capsule structure. Both capsule stability and permeability can to a great extent be controlled by the polycation exposure 13, 16, 17, 18. Alginate‐PLL based capsules were the first to be tested in clinical trials of encapsulated human islets in 1994 [19] and alginate‐PLO based capsules have been tested more recently 20, 21 (Table 1). However, the pro‐inflammatory properties of polycations have been linked to a fibrotic response toward the capsules upon transplantation, also after the addition of a second coating layer of alginate 22. The attachment of host cells on the surface of the capsules is believed to limit the diffusion of oxygen and nutrients to the encapsulated islets and lead to nonfunctioning grafts 23, 24. Hence, newer capsule developments are based on using the alginate solely without the polycation layer. Progress in newer capsule designs has led to two additional encapsulated human islet transplantations to diabetic recipients (Table 1) 25, 26. Although the studies conclude with a safe procedure, the efficacy of the transplanted graft has been limited. Hence, challenges still remain in making capsules that support graft function.

**Table 1:** Overview of capsules used in encapsulated human islet transplantations to diabetic recipients

Capsule composition	Number of patients	References
Alginate‐poly‐l‐lysine‐alginate	1	19
Alginate‐poly‐l‐ornithine‐alginate	4	20, 21
Barium‐alginate	4	25
Calcium/Barium‐alginate	1	26

## Important Capsule Properties

**Figure 2:**
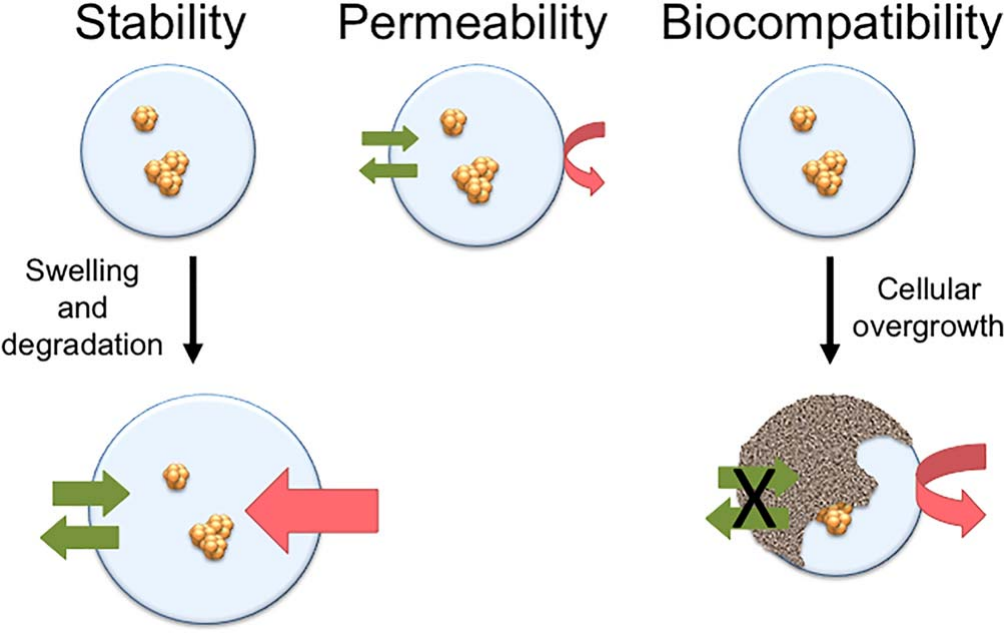
Important capsule properties are stability, permeability and biocompatibility. These properties are interconnected as the capsule permeability will increase with capsule destabilization through swelling and this may lead to loss of immune protection. The attachment of cells on the capsule surface leads to limited access of nutrients and oxygen for the encapsulated cells.

The challenges of supporting graft function may be linked to some capsule properties that are believed to be of major importance. This includes (a) stability, as the capsules need to protect the graft over a prolonged period of time, (b) permeability to allow the diffusion of nutrients and oxygen and limit the access of the immune system and (c) biocompatibility, that in this setting is mostly connected to avoiding a fibrotic response (Fig. 2). A thorough review on capsule properties is given in 27.

Stable alginate beads without a polycation layer can be made by careful selection of alginate and gelling ion. Alginate is composed of β‐d‐Mannuronic acid (M) and its C5 epimer α‐l‐Guluronic acid (G) that is 1‐>4 linked via glycosidic linkages. The M and G monomers are found in M‐blocks, G‐blocks, and MG‐blocks of various lengths. The sequential arrangement of M and G, together with the molecular weight, determines to a great extent the properties of the alginate, and consequently the properties of the gel beads 28. Originally, alginate beads were made by gelation with calcium ions. Calcium is historically known to cross‐link G‐blocks in the alginate, however, recent results show that also MG‐blocks participate in the cross‐linking with calcium 29. Hence, alginates with a high content of G, situated in either G‐blocks or MG‐blocks, form relatively stable beads with calcium. However, upon the removal of polycation followed a need to increase the stability of the alginate beads. Alginate beads are vulnerable toward destabilization by the depletion of calcium ions at physiological conditions due to chelating compounds such as phosphates, and also by the exchange with sodium ions. More stable calcium‐alginate beads can be made by enzymatic modification of the alginate to contain solely G‐ and MG‐blocks 30. Though, more commonly used is the addition of barium ions to the gelling solution that also may result in more stable beads. Barium ions bind strongly to the G‐blocks in the alginate and to some extent to the M‐blocks 31, and incorporate shorter G‐blocks in the cross‐links upon gelation compared to calcium 32. In fact, the addition of 1 mM barium ions to a solution of 50 mM calcium ions has a great impact on the stability of alginate beads made of alginate with a high content of G‐blocks 31. Such Ca/Ba‐alginate beads have been shown to protect human islets in a diabetic mouse model for up to 220 days 33. When transplanted to a diabetic patient, positive c‐peptide was detected until 3 months post‐transplantation 26. Also, higher barium ion concentrations (e.g., 10 mM and 20 mM) have been used for encapsulation, without the use of calcium 24, 25, 34. Using higher barium concentration poses the risk of leakage of potential toxic barium ions to the recipients. The accumulation of barium in blood and femur bone in mice has been linked to increased barium concentrations in implanted alginate beads 35. Hence, care should be made for clinical transplantation regarding the selection of alginate and gelling ions, as well as washing procedures.

Polycation containing capsules were shown to limit the ingress of the antibodies as well as the larger cytokines (e.g., TNF‐α) and the permeability can be tuned based on the polycation type and exposure time 17. However, for the permeability of small cytokines, nitric oxide, and free radicals, both from the islets as well as from the host, it is challenging to limit the diffusion through the capsules by pore size as the absolute request of the capsules is to allow the diffusion of glucose and insulin, as well as some of the larger proteins, like transferrin 14. Alginate beads, without the polycation, are more open structures that do not exclude the cytokines or complement factors. It is not known what permeability is needed for the capsules to function as an immune barrier, but it may seem like the most important function of immune isolation lies in the avoidance of cell‐to‐cell contact between the graft and the host, as there are now several reports of the function of both allo‐ and xeno grafts in alginate beads without a polycation coating in mouse models 33, 34, 36, 37. Alginate beads of different permeability toward antibodies (e.g., IgG) can be made with the selection of alginate composition and gelling ions 30, 31, and barium gelled alginate beads are shown to protect transplanted allograft in mice despite antibody response of IgG and IgM 38.

In small animal models, for example, mice and rats, the alginate beads are in general shown to promote minimal fibrotic overgrowth. However, there may still be a problem connected to the fibrosis of alginate beads in larger animal models such as monkeys and humans. Although not promoting any detectable inflammatory responses in human blood 39, 40, the two clinical transplantations of human islets in Ba‐alginate and Ca/Ba‐alginate beads 25, 26 may indicate a continued problem of fibrosis, even when omitting the polycation. Fibrosis is also seen in one immune competent mouse strain (C57Bl6 mice), which is consequently used for the evaluation of capsule biocompatibility where also differences between alginate beads can be observed 41, 42, 43. In this strain of mice, alginate beads with a high G content have been shown to promote fibrosis, whereas for alginate beads with a lower G content fibrosis was not observed, regardless of the gelling ions used 43. However, the low stability of the beads consisting of alginate with a low G content limits the immediate use of these alginates for encapsulation. This challenge may be met by enzymatically tailored alginates that can be made very stable with a low G content 30. Very recently, a positive effect on fibrosis has been shown by using chemically modified alginate in the alginate beads in a mixture with nonmodified alginate 44. Positive effects on limiting fibrosis were seen with structurally diverse chemical substituents. As the alginates were substituted on the carboxylic groups, the removal of charge upon substitution is one general effect, resulting in a lower charge density of the alginate. However, the differences in responses for the varying substituents clearly show that the type of molecule matters for the response. Very recent results also show a dependency of size, as larger alginate beads of about 1.5 mm in diameter had a significantly lower fibrotic response than the conventional size of about 0.5 mm in diameter 24. Less fibrosis on larger capsules was shown both in C57Bl6 mice and in monkeys. Although no negative effect on islet function was reported as a result of increasing capsule size, questions remain regarding a possible limited oxygen supply for the encapsulated islets in general as well as with increasing capsule size.

## The Inner Life of the Capsule—Oxygen Supply and Microenvironment

Although no difference has been detected on the transcriptome of microencapsulated islets versus nonencapsulated islets 45, concerns have been raised regarding the islets' access to oxygen and nutrients. The oxygen concentration in alginate microcapsules has been estimated to be constant up to about 200 μm into the capsule 46. Hence, the ideal capsule size for maximum oxygen access would be less than 400 μm. The spherical shape and large surface to volume ratio, makes microcapsules attractive for the access of oxygen and nutrients compared to larger devices and devices of other geometries. Islets in their natural habitat are highly vascularized. Hence, lack of vascularization and potential hypoxia is also an issue without capsules, however with capsules the potential ingrowth of blood vessels into the islets is physically hindered by the capsule. For short‐term (acute) hypoxia in vitro, no difference on viability or function was seen for encapsulated versus nonencapsulated human islets 47. However, culture of encapsulated islets under low oxygen conditions shows loss of viable tissue and necrotic cores 48. To counteract the lowering of oxygen tension, perfluorocarbon emulsion 48and CaO_2_ 49 have been suggested as coencapsulated sources of oxygen. Although the need for increased oxygen supply is debated for microencapsulated pancreatic islets, indeed, positive effects have been seen on islet viability in a clinical transplantation of a macrodevice, where oxygen was infused to the encapsulated islets on a daily basis 50. Also, pretreatment of the transplantation site to induce vascularization has been shown to increase the function of the graft without capsules 51, and strategies for increased vascularization to the graft are being pursued 52, 53.

A bottleneck in islet transplantation has been the access to enough insulin‐producing tissue, where also porcine islets have been suggested as a potential source 54. Recent break‐through in stem cell differentiation toward insulin‐producing cells as well as induced pluripotent stem cells (iPSC), gives promising perspectives of obtaining cells from human sources. Functional beta cells have been produced in vitro from human embryonic stem cells (hESC) 5, 6, and very recently from iPSCs from diabetic patients 55. With cells form human sources, and potentially from the patients' own cells, it is assumed that the host immune response toward the graft will be largely eliminated. However, as type 1 diabetes is an ongoing autoimmune disease, capsules may still be needed to avoid the destruction of the new beta cells from the immune system. For the encapsulation of stem cell derived tissue, there is no obvious reason to change the design criteria of the capsules, except for a possible change in criteria for capsule permeability. ViaCyte is currently in Phase 1/2 clinical trial to test the safety and efficacy of their macroencapsulated pancreatic progenitors derived from hESCs. The ViaCyte team has shown the development of pancreatic islet like tissue in their macrodevice in mice 4, where micro vessels were formed around and partly into the device. This is one of the first embryonic stem cell derived therapies to be tested in the clinic. For the recently shown full differentiation in vitro 5, 6, the differentiation protocols are complex with respect to culture conditions as clusters and suspensions and varying glucose and oxygen levels have been used in addition to various growth factors 5, 6. A positive effect of encapsulation has been shown for the development of neonatal porcine islets into mature islets upon culture in serum‐containing media 56. This may be partly due to the hindering of aggregation of clusters upon culture, as has also been shown for human pancreatic progenitors when cultured in alginate beads 57. hESC have been differentiated to definitive endoderm in alginate capsules 58. For pancreatic progenitors, no effect on differentiation was seen when extracellular matrix (ECM) components (proteins and peptides) were added to the capsule composition 57. Although no effect was seen in the previous study, the design of the capsule microenvironment to mimic the natural microenvironment has drawn recent attention 59and positive effects have been shown on beta cell lines 60 as well as on human islets 61. The use of heparan sulfate in the differentiation protocol of pancreatic islets 5is interesting in this perspective as this is a natural component of the ECM. Sulfated alginate mimics the structure of heparan sulfate and the function related to the binding of growth factors 62, 63 may be an interesting alternative to chemical modification of alginate capsules, both related to the microenvironment for the encapsulated cells as well as for the reduction of fibrosis 64.

## Conclusion

In conclusion, transplantation of human islets in alginate‐based capsules have been clinically evaluated in four small trials and are in general recognized as safe. However, limited graft function has been connected to the observed fibrosis on the capsules limiting the access of oxygen and nutrients to the encapsulated islets. Although the problem of fibrosis has been reduced as a function of avoiding the polycation in the capsules, fibrosis seems to still be a problem. The mechanisms of fibrosis on alginate beads are still not elucidated. However, very recent positive findings regarding increased size of capsules and chemical modifications of the alginate where reduced fibrosis is seen also in monkeys, make an optimistic view of the future of encapsulated islet transplantation. Importantly, the very recent developments of insulin‐producing cells from stem cells, gives the transplantation of encapsulated insulin‐producing tissue a very promising outlook both long‐term and in the near future. As type 1 diabetes is an autoimmune disease, the capsules may still be needed regardless of cell source for a protection of the graft from the destruction from the recipient's own immune system.

## Author Contributions

B.L.S. and G.S.‐B.: conception and design, financial support, manuscript writing, final approval of manuscript; A.E.C: manuscript writing, final approval of manuscript.

## Disclosure of Potential Conflicts of Interest

The authors indicate no potential conflicts of interest.
